# A new molecular marker including parts of conservative histone H3 and H4 genes and the spacer between them for phylogenetic studies in dragonflies (Insecta, Odonata), extendable to other organisms

**DOI:** 10.18699/vjgb-25-94

**Published:** 2025-10

**Authors:** A.V. Mglinets, V.S. Bulgakova, O.E. Kosterin

**Affiliations:** Institute of Cytology and Genetics of the Siberian Branch of the Russian Academy of Sciences, Novosibirsk, Russia; Institute of Cytology and Genetics of the Siberian Branch of the Russian Academy of Sciences, Novosibirsk, Russia; Institute of Cytology and Genetics of the Siberian Branch of the Russian Academy of Sciences, Novosibirsk, Russia Novosibirsk State University, Novosibirsk, Russia

**Keywords:** histone repeat, histone H3, histone H4, intergenic spacer, Odonata, dragonflies, insects, molecular marker, phylogenetic studies, гистоновый повтор, гистон Н3, гистон Н4, межгенный спейсер, Odonata, стрекозы, насекомые, молекулярный маркер, филогенетические исследования

## Abstract

In spite of recent substantial progress in genomic approaches, there is still a need for molecular markers convenient for Sanger sequencing and providing good phylogenetic reconstructions at short evolutionary distances. A new molecular marker, the histone H3–H4 region, containing partial coding sequences of the genes for histones H3 and H4 and the non-coding spacer between them, is proposed. This marker is potentially useful for molecular phylogenetic studies at the generic, species, and even intra-species level in insects and some other organisms, even from other phyla. The highly conserved histone-coding sequences ensure the universality of primers and the ease of primary alignment, while the highly variable non-coding spacer provides enough variation for analyses at short evolutionary distances. In insects, the histone genes reside in the histone repeat which is tandemly repeated in dozens to hundred copies forming the so-called histone cluster. This ensures a high concentration of the template for the marker in genomic DNA preparations. However, the order and orientation of the histone genes in the histone repeat is variable among orders, which puts some limitations on the use of the proposed marker. The marker efficacy is hereby shown for Odonata (dragonflies and damselflies), where it provided good resolution at the family, genus and species levels. The new marker also provided an interesting pattern in the relationship of two Sympetrum species, S. croceolum and S. uniforme, showing the sequences of the latter as a branch nested among those of the former. The same combination of the proposed original primers should also work in Diptera

## Abstract (ru)

Несмотря на значительный прогресс в геномных исследованиях, до сих пор существует потребность в молекулярных маркерах, удобных для секвенирования по Сэнгеру и позволяющих делать филогенетические реконструкции на коротких эволюционных дистанциях. Предложен новый молекулярный маркер, район генов гистонов Н3 и Н4, содержащий части кодирующих последовательностей генов гистонов Н3 и Н4 и некодирующий спейсер между ними. Он может быть удобен для молекулярно-филогенетических исследований на родовом, видовом и даже внутривидовом уровне у насекомых и некоторых других групп, в том числе из других типов. Высококонсервативные кодирующие последовательности гистоновых генов обеспечивают универсальность праймеров и однозначность первичного выравнивания, тогда как вариабельный некодирующий спейсер содержит достаточно изменчивости для анализа на коротких эволюционных дистанциях. У насекомых гены гистонов находятся в гистоновом повторе, тандемно повторенном в десятках или сотнях копий в так называемом гистоновом кластере. Это обеспечивает высокую концентрацию матрицы для нового маркера в препаратах геномной ДНК. Однако порядок и ориентация конкретных генов у разных отрядов изменчива, что налагает определенные ограничения на использование предлагаемого маркера. В нашей работе эффективность маркера продемонстрирована на материале отряда стрекоз (Odonata), где он хорошо разрешил семейства, роды и виды. Новый маркер также выявил интересный паттерн взаимоотношения двух видов рода Sympetrum, S. croceolum и S. uniforme, показав последовательности последнего в качестве ветви среди последовательностей первого. Та же комбинация разработанных нами оригинальных праймеров будет работать и в отряде двукрылых (Diptera).

## Introduction

Analysis of DNA variation is a powerful tool in reconstructing phylogenetic history of living creatures, so the use of molecular methods has provided a profound progress in phylogenetic analysis, with applications in taxonomy, paleobiology, paleogeography and evolutionary theory. Particular sequences used for this purpose, traditionally called ‘phylogenetic markers’, differ in their rate of fixation of mutations thus permitting phylogenetic resolution at different time scales, with resolution of most recent divergences being possible with most variable markers; for those applied to Odonata see Y.C. Cheng et al. (2018)

The modern next-generation high-throughput sequencing and genomic approaches offer ample phylogenetic data (for examples in Odonata, see Futahashi et al., 2015; Bybee et al., 2021; Kohli et al., 2021), potentially useful for analysis even at short evolutionary distances, but are expensive and more demanding in sample preparation. Although the prices are getting lower, these technologies still remain unaffordable for many researchers in countries which harbour the richest biodiversity. Therefore, there is still a need for easily analysed and cheap nuclear markers based on Sanger sequencing which would provide good resolution at the species level and could be useful at least for fast preliminary tests revealing the evolutionary history of populations, subspecies and closely related species. Besides, such markers make it possible to analyse regular collection specimens, not specially collected for DNA analysis

Non-coding sequences, the variability of which is mainly determined by physical properties of DNA replication, are useful candidates for variable phylogenetic markers. Their use is limited by possibility of working out universal primers, which could be achieved by involvement of bordering conserved sequences. Besides, non-coding sequences demonstrate a high rate of indels, which bring about difficulties as to their alignment. Of such markers, the so-called ITS region including internal spacers between the conserved ribosomal RNA genes, ITS1 and ITS2, is the most popular among nuclear markers; for its use in Odonata, see Hovmöller and Johansson (2004), Dumont et al. (2010), Karube et al. (2012), Schneider et al. (2023). The highly repetitive nature of the nucleolus organiser provides an advantage of high concentration of the template in preparations of genomic DNA and a disadvantage of possible heterozygosity as well as cis-heterogeneity between individual repeat copies (Hovmöller, Johansson, 2004).

Recently, a useful approach has been proposed, focusing on introns of nuclear genes (Ferreira et al., 2014). The primary structure of introns has scarce adaptive constraints save mutations affecting splicing. At the same time, the bordering exons are usually conserved enough to allow for universal primers design. These markers have a disadvantage of low concen tration of template genomic DNA, since the genes involved are unique and present in the genome only in two copies, so they may be less readily amplifiable from specimens with somewhat degraded DNA as compared to the highly repeated sequences of the ITS region

In the present work, we propose and test the usability of a new phylogenetic marker, the spacer between the genes of the conservative core histones H3 and H4 and partial coding sequences of these histones, which we designate as the histone H3–H4 region. It resembles the popular ITS region mentioned above

**Fig. 1.**
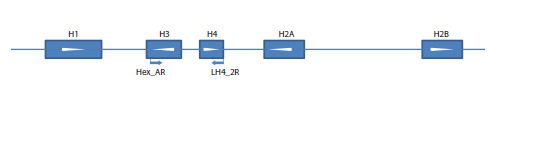
Positions of the primers used to amplify the histone H3–H4 region in the histone repeat as exemplified by a fragment of the assembly of the Ischnura elegans genome (NW_025791746). White arrowheads indicate the direction of transcription

In animals, the genes for five histones (H1, H2A, H2B, H3, H4) are included into the so-called histone repeat, tandemly repeated copies of which form the histone cluster (Eirín-López et al., 2009). Several important circumstances should be noted in this respect:

(i) Histones H3 and H4 are among the most conserved proteins in eukaryotes (Stein et al., 1984; Doenecke et al., 1997; Eirín-López et al., 2009), so their coding sequences allow primers of very broad applicability across taxonomic groups.

(ii) In insects, genes of these two histones are disposed in the histone repeat relatively close to each other (Eirín-López et al., 2009). The order of histone genes and orientation of their reading frames is variable among different insect orders. For instance, their order is H1, H3, H4, H2A, H2B in Drosophila melanogaster (Goodenough, 1984: p. 304; Eirín-López et al., 2009) and in all Odonata species tested in this work (Fig. 1). Although this variation may also take place at lower taxonomical levels and is sometimes observed even in different copies of the histone repeat in the same chromosome, in all species of Odonata tested by us (see below) we obtained a PCR product using the same primer pair matching histones H3 and H4. So the original primers we suggest are useful at least across dragonflies and damselflies

(iii) The spacer between H3 and H4 histone genes is non-coding and therefore is expected to undergo neutral evolution, hence being a kind of molecular clock

(iv) Insects have hundreds of copies of the histone repeat (Stein et al., 1984; Solovyev et al., 2022), which facilitates amplification from total genomic DNA preparations, but may also bring about problems related to cis- (withincluster) and trans- (allelic, between homologous chromosomes) heterogeneity.

(v) Most insects have only one histone cluster, whereas in some of them and in other animal groups there is a number of paralogous clusters (Eirín-López et al., 2009). A single histone cluster is an advantage since this avoids transcluster heterogeneity which would complicate an analysis

To develop and test the marker, we chose the order Odonata and designed original primers which worked in all tested species. We tested its resolution at different taxonomical levels, by sequencing amplicons from representatives of different families (Calopterygidae, Coenagrionidae, Aeshnidae, Gomphidae, Corduliidae, and Libellulidae), from several species of some genera and from a number of specimens of some Sympetrum spp. The latter involved a series of three species from the same danae species group (Pilgrim, Dohlen, 2012), namely S. croceolum (Selys, 1883), S. danae (Sulzer, 1776) and S. uniforme (Selys, 1883), including those collected in the same populations.

## Materials and methods

Material. The specimens of S. croceolum, S. danae, S. flaveolum (Linnaeus, 1758), and S. uniforme were preserved in 96 % ethanol, other specimens were treated overnight with acetone and then dried out. The species and specimens from which the histone H3–H4 region was sequenced in the course of this study and the GenBank accession numbers of these sequences are enumerated (in parentheses) in the Table

**Table 1.**
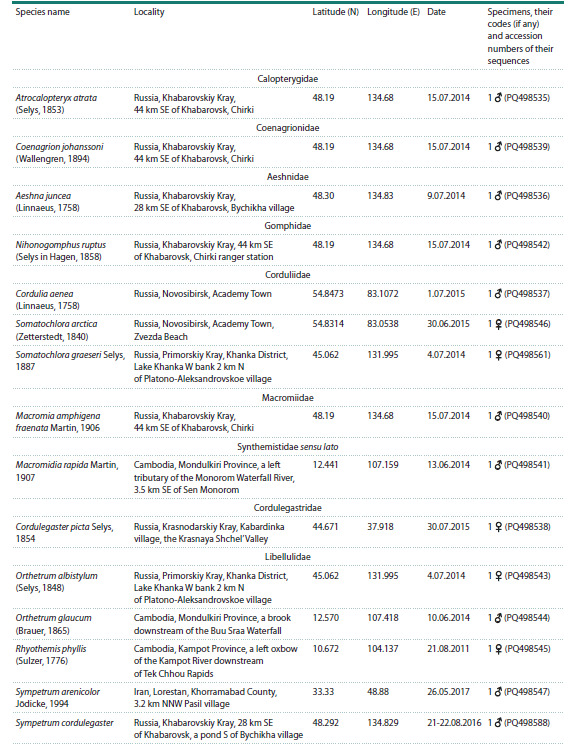
Species (by families) and specimens sequenced for the histone H3–H4 region, their origin and the GenBank accession numbers of the sequences. Coordinates are given in decimal degree format

**Table 1end.**
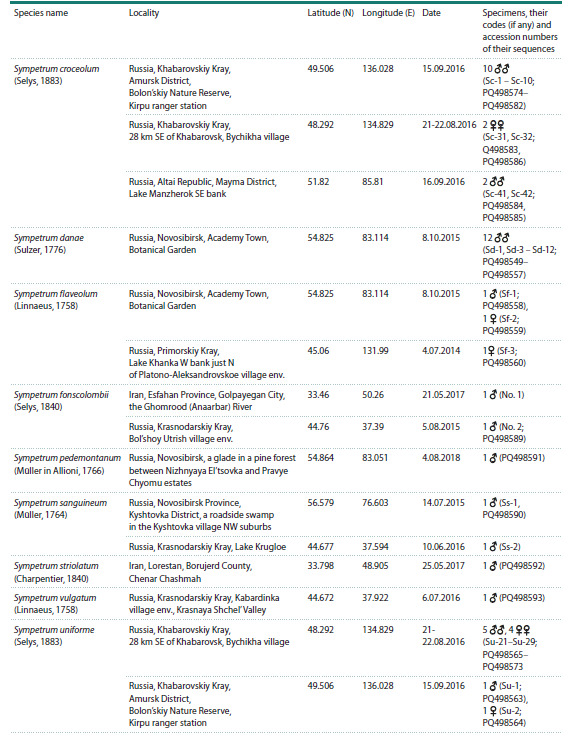
Table 1end.

Sequences from public databases. To expand our sample, we downloaded sequences of the H3–H4 region from the Whole Genome Sequence datasets available in public databases of 16 more Odonata species: Hetaerina americana (Fabricius, 1798), H. titia (Drury, 1773) (Calopterygidae), Argia fumipennis (Burmeister, 1839), Ceriagrion tenellum (De Villers, 1789), Ischnura elegans, I. senegalensis (Rambur, 1842) Pseudagrion microcephalum (Rambur, 1842), Pyrrhosoma nymphula (Sulzer, 1776) (Coenagrionidae), Platycnemis pennipes (Pallas, 1771), Prodasineura notostigma (Selys, 1860) (Platycnemididae), Tanypteryx hageni (Selys, 1879), Tachopteryx thoreyi (Selys, 1889), Uropetala carovei (White in Dieffenbach, 1843) (Petaluridae), Brachytron pratense (Müller, 1764) (Aeshnidae), Pachydiplax longipennis (Burmeister, 1839), Pantala flavescens (Fabricius, 1798), (Libellulidae).

**Fig. 2.**
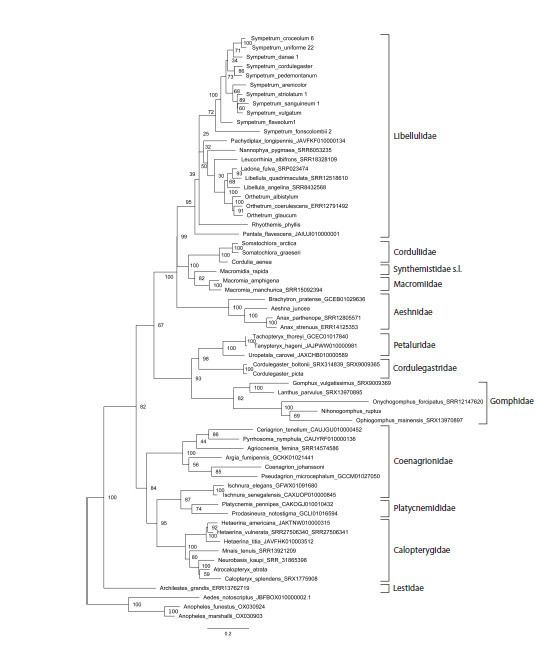
Phylogenetic tree of the studied species of Odonata reconstructed with Maximum Likelihood method from the histone H3–H4 region sequences. Bootstrap values are shown at the respective nodes. Three species of Diptera, Culicidae serve as the outgroup.

**Fig. 3.**
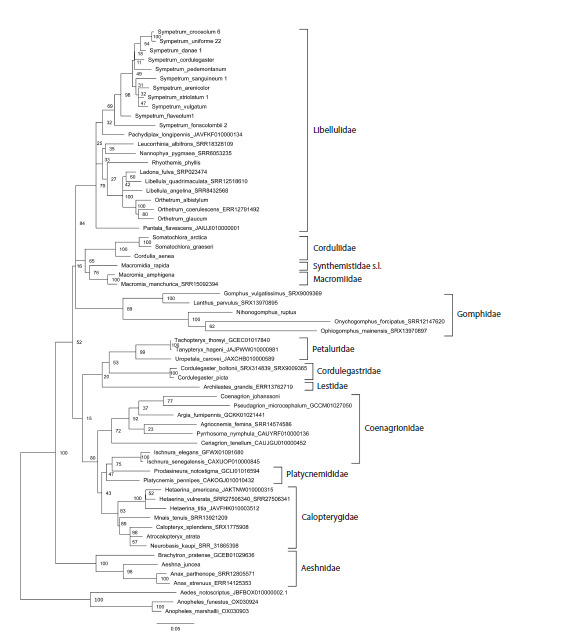
Phylogenetic tree of the studied Odonata species reconstructed with the Maximum Likelihood method from fragments of the coding sequences of histone H3 and histone H4 genes involved into the proposed ‘histone H3–H4 region’ marker. Bootstrap values are shown at the respective nodes.

Besides, the histone H3–H4 region was assembled from SRA archives available at GenBank, with the use of the MIRA software (Chevreux et al., 1999) for the following 20 species: Archilestes grandis (Rambur, 1842) (Lestidae), Calopteryx splendens (Harris, 1780), Hetaerina vulnerata (Hagen in Selys, 1853), Mnais tenuis (Oguma, 1913), Neurobasis kaupi (Brauer, 1867) (Calopterygidae), Agriocnemis femina (Brauer, 1868) (Coenagrionidae), Anax parthenope (Selys, 1839), A. strenuus (Hagen, 1867) (Aeshnidae), Gomphus vulgatissimus (Linnaeus, 1758), Lanthus parvulus (Selys, 1854), Onychogomphus forcipatus (Linnaeus, 1758), Ophiogomphus mainensis (Packard in Walsh, 1863) (Gomphidae), Cordulegaster boltonii (Donovan, 1807) (Cordulegastridae), Macromia manchurica (Asahina, 1964) (Macromiiddae), Ladona fulva (Müller, 1764), Leucorrhinia albifrons (Burmeister, 1839), Libellula angelina (Selys, 1883), L. quadrimaculata (Linnaeus, 1758), Nannophya pygmaea (Rambur, 1842), Orthetrum coerulescens (Fabricius, 1798) (Libellulidae). The accession numbers of the database entries used as sources of these sequences are indicated at species names in Figures 2 and 3

Primer design. We designed 14 original primers to match different parts of insect histone genes coding for H1, H2B, H3, H4, comprising the histone gene cluster. At the start of the present work we did not know the precise order and orientation of the histone genes in the cluster, so we tested different primer combinations to select primer pair(s) which would produce an amplification product containing the fragments of the genes of histone H3 and H4 and the spacer between them. We found out that the pair of primers Hex_AR matching the 3ʹ portion of the H3 gene (in the orientation opposite to that of transcription) and LH4_2R matching the 3ʹ portion of the H4 gene (also in the orientation opposite to that of transcription) (Fig. 1) produced the expected product, indicating that the H3 and H4 genes were oriented anti-parallel as to their reading frames, with 5ʹ ends of their coding chains oriented towards each other (Fig. 1). The primer sequences are as follows:

Hex_AR: 5ʹ atatccttgggcatgatggtgac (forward) LH4_2R: 5ʹ ttaaccgccgaaaccgtacagggt (reverse).

The primer LH4_2R, matching the coding region of the histone H4 of the moth Bombyx mori (Linnaeus, 1758) (Lepidoptera: Bombycidae) (GenBank accession AADK01010708), was worked out in the course of our previous study of the variation of the histone H1 gene in some Lepidoptera (Solovyev et al., 2015), although this particular primer was not mentioned in the cited work and is published here for the first time.

The Hex_AR primer was worked out to match the coding sequences of the histone H3 gene of Ophiogomphus severus (Hagen, 1874) (Odonata: Gomphidae) taken from GenBank (accession AY125228).

The coding sequences of histones H3 and H4 are well conserved (Stein et al., 1984; Doenecke et al., 1997; Eirín-López et al., 2009), so the primers worked out to match a particular sequence, one of which was from Odonata and the other from Lepidoptera, worked well for all tested species of Odonata

DNA isolation, sequencing and analysis. Dragonfly legs were homogenized in a mortar in 0.2 ml of isolation buffer (0.1 Tris-HCl, pH 8.0; 0.05 M EDTA; 1.25 % SDS; 0.5 M NaCl) with Al2O3 as grinding particles, then mixed with 0.8 ml of the same buffer. The mixture was incubated for 1 h at 55 °C, then 350 μl of 5 M potassium acetate was added, the mixture was incubated for 30 min on ice and centrifuged at 16.1 g for 10 min. The supernatant was transferred to fresh tubes, mixed with 0.6 ml of isopropanol, incubated at room temperature for 1 h and centrifuged at 12.2 g for 10 min. The precipitate was washed twice with 0.1 ml 70 % ethanol with subsequent centrifugation at 12.2 g for 5 min, dried at 50 °C for 5 min and dissolved in 50 μl of deionized water.

PCR reaction was carried out in a volume of 20 μl with 2 μl of 10× ammonium sulphate buffer, 2 μl of 25 mM MgCl2, 0.3 μl of the Hot Start Tаq polymerase produced by SIBENZYME company, Novosibirsk (5 U/μl), 0.15 μl BSA (10 mg/ml), 1 μl of forward and reverse primers (10 pM) each, 2 μl of 2 mM dNTPs, 2 μl of diluted DNA (20–60 ng) and 10.55 μl of deionised water. For PCR, BIO-RAD MyCycler thermal cycler was used, with the reaction parameters as follows: denaturation at 95 °C for 3 min followed by 32 cycles including denaturation at 94 °C for 30 s, annealing at 55 °C for 25 s, elongation at 72 °C for 45 s. The PCR products were purified with Invisorb® Spin Filter PCRapid Kit and Sanger sequenced using Big Dye Terminators version 3.0 or 1.1 at SB RAS Genomic Core Facility

Raw trace files were visualized and translated into nucleotide sequences with the use of the Gap4 software (Staden et al., 2003). The sequences were aligned with ClustalW (Larkin et al., 2007) using the MEGA 6.0 software package (Tamura et al., 2013) with default parameters. For a separate analysis of the non-coding spacer between the histone H3 and H4 genes, the relevant part of the alignment of the entire histone H3–H4 region was used, since separate alignment of the spacer sequences as such is less reliable

**Fig. 4.**
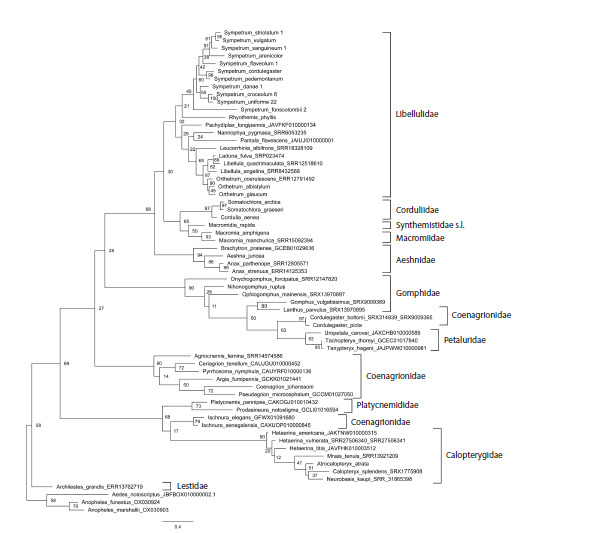
Phylogenetic tree of the studied Odonata species reconstructed with the Maximum Likelihood method from the intergenic spacer between histone H3 and histone H4 genes. Bootstrap values are shown at the respective nodes.

The phylogenetic relationships were reconstructed with the Maximum Likelihood method using MEGA 6.0, with Kimura 2-parameter substitution model, as default in the package; rate among sites: gamma-distributed with invariant sites. Bootstrap values from 100 replications were calculated. The sequences of the histone H3–H4 region of three species of Diptera, Aedes notoscriptus (Skuse, 1889), Anopheles funestus (Giles, 1900) and A. marshalli (Theobald, 1903) from GenBank, were used as the outgroup for the order Odonata-wide phylogenetic reconstruction (Figs 2–4), because in Diptera we found the same order and orientation of the genes of histones H3 and H4

The uncorrected p-distances between different alleles of the histone H3–H4 regions within species of Sympetrum were calculated with the MEGA 6.0 software (the entire matrix is not shown).

## Results

The histone H3–H4 region was successfully amplified with the above suggested primer pair and sequenced from DNA isolated from specimens of Odonata enumerated in the Table, 59 individuals of 24 species. Together with the 36 sequences adopted from public databases, this comprised a sample of 95 sequences of 60 species

The sequences of the histone H3–H4 region contained parts of the conservative coding sequences of the genes of histones H3 (351 b.p.) and H4 (288 b.p.) and the spacer between them of a variable length of about 250 b.p. All substitutions revealed in the coding sequence fragments were synonymous except for the substitution T ˃ A in the first position of the second codon of the histone H4 gene, which changes threonine to serine, in both sequenced specimens of S. fonscolombii (Selys, 1840) (not shown). As expected, the sequences of bordering coding sequences were unambiguously aligned. At the same time the spacer is expectedly hyper-variable and exhibits a high rate of indels, so its alignment was much less certain and retained some ambiguity. The alignment involving one sequence per each studied species, including the outgroup, used for reconstruction of the phylogenetic tree of Figure 2, was 1019 b.p. long and had 583 (57 %) variable sites, 529 (52 %) parsimoniously informative sites and 42 (4 %) singletons. These numeric estimates, however, are conventional and should be taken with caution because of uncertainty of the alignment of the non-coding sequence of evolutionary distant species

One specimen of S. sanguineum (Müller, 1764) (Ss-2), one specimen of S. fonscolombii (No. 1), and one specimen of S. uniforme (Su-23) appeared to be heterogeneous containing reads with and without deletion of a number of nucleotides in the spacer. One of those indels found in S. sanguineum (Ss-2) concerned just one base pair; so we were able to infer both sequence variants from the chromatogram but used for further analysis only one of them, chosen randomly. Indels found in the other two species were longer, about 5 and 10 b.p. Although the sequences beyond the deleted region could be reconstructed, we preferred to exclude these specimens from further analyses.

In some positions, the chromatograms revealed two peaks of comparable height suggesting within-specimen heterogeneity for nucleotides occupying these positions. Those positions reflected either heterozygosity for different alleles or cisheterogeneity for the histone repeat along a histone cluster, quite expectable in the case of repeated units. Such positions made uncertain the exact number of unique alleles found in a species. A few chromatograms did not resolve nucleotides in a number of positions adjacent to the primers; we nevertheless involved the shortened, well resolved sequences into phylogenetic reconstructions

First, we reconstructed a phylogenetic tree based on the sequence of the H3–H4 region from one representative of each involved species, both newly sequenced and available or reconstructed from public databases (Fig. 2). The tree was rooted with the sequences of mosquitoes Anopheles and Aedes used as the outgroup. The overall tree topology well corresponded to the family system of Odonata (Dijkstra et al., 2013), except for the odd position of the genus Ischnura, which is attributed to Coenagrionidae but clustered with Platycnemididae, although with a weak bootstrap support of 83.

Our tree included 10 currently recognised families of Odonata (Dijkstra et al., 2013) represented by several species. Seven of them were revealed as monophyletic clades well supported by high bootstrap values: Libellulidae (95), Corduliidae (100), Macromiidae (100), Aeshnidae (100), Petaluridae (100), Cordulegastridae (100) and Calopterygidae (100). Two families appeared monophyletic with weak support: Gomphidae (82) and Platycnemididae (74). The cluster of Coenagrionidae without Ischnura had the highest support of 100. Even representatives of the three families (Corduliidae, Macromiidae and Synthemistidae s. l.), previously considered in the family Corduliidae in the broad sense, also grouped in a cluster with the maximum support of 100.

Archilestes grandis is the only involved representative of Lestidae, the family considered to retain most plesiomorphic characters among Odonata (Dijkstra et al., 2013). Hence its position as the most basal branch of Odonata was rather expected. If we were to exclude this branch formally attributed to Zygoptera, both suborders Anisoptera and Zygoptera would appear monophyletic but weakly supported (67 and 84, respectively).

The tree of Figure 2 includes nine genera represented by more than one species. Seven of them (Orthetrum Newmann, 1833, Somatochlora, Macromia Rambur, 1842, Anax Leah in Breuster, 1815, Cordulegaster Leah in Breuster, 1815, Ischnura Charentier, 1840, Hetaerina Hagen in Selys, 1853) had the highest support of 100. The genus Sympetrum, represented by 11 species, formed a monophyletic cluster with weak support (72). However, if we were to exclude the problematic (see below) divergent species S. fonscolombii, the remained 10 species would cluster together with the maximum support of 100. The genus Libellula Linnaeus, 1758 would become monophyletic but weakly supported (68) if we were to assume Ladona Needham, 1897 to be its synonym, as it is often considered

It was interesting to evaluate separate inputs into this phylogenetic resolution of the histone coding sequences and spacer, so we reconstructed phylogenetic trees based on these two components separately (Figs 3, 4). In both trees, terminal branches uniting close species or genera are mainly well supported. The support of families is somewhat lower than in the tree based on the entire histone H3–H4 region (Fig. 2), with the values in the tree based on the concatenated coding sequences of both histone genes (Fig. 3) being in general higher than in the tree based on the non-coding spacer (Fig. 4). The principal topology of the tree based on the spacer sequences (Fig. 4) remained similar to that of the tree based on the entire H3–H4 region (Fig. 2), but is not supported. The overall topology with respect to positions of families of the tree based on the coding sequences (Fig. 3) is different, does not reflect dichotomy for the two suborders and is even less supported than in the spacer tree (Fig. 4). This can be attributed to saturation of conservative histone gene sequences by synonymous substitutions at long evolutionary distances. Altogether, we may conclude that both parts of the histone H3–H4 region have their input into its resolving power, but the best result is produced by the two parts taken together.

**Fig. 5.**
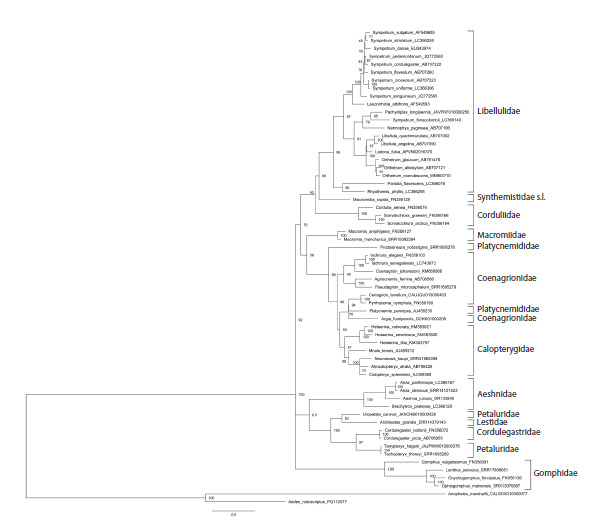
Phylogenetic tree of the species of Odonata as in Figure 2 (with two omissions) reconstructed with the Maximum Likelihood method from the ITS region sequences adopted from GenGank. Bootstrap values are shown at the respective nodes. Two species of Diptera, Culicidae serve as the outgroup

As stated above, the phylogenetic marker proposed here, the histone H3–H4 region, is similar to the popular ITS region containing rRNA genes and two non-coding spacers between them. To compare phylogenetic resolution of these two markers, we reconstructed a phylogenetic tree based on the ITS region sequences adopted from GenBank, which contains the same species except for N. ruptus and S. arenicolor (Fig. 5). The alignment of these sequences was 1048 b.p. long and had 878 (84 %) variable sites, 529 (71 %) parsimoniously informative sites and 118 (11 %) singletons. These data are also affected by the ambiguity of the alignments of the noncoding spacers as those for the histone H3–H4 region and should be taken with caution. The ITS region contained a somewhat greater share of parsimoniously informative sites than the histone H3–H4 region, 71 vs 52 %. However, the ITS marker appeared substantially inferior in resolving odonate families as compared to the histone H3–H4 region, as seen in the phylogenetic tree reconstructed from the ITS region (Fig. 5). This tree contains a number of awkwardly placed species. The zygopteran Archilestes grandis occurs among Anisoptera where it clusters with U. carovei, which in turn does not cluster with the two other Petaluridae. P. pennipes does not cluster with the second Platycnemididae, P. notostigma, but occurs among representatives of Coenagrionidae. The branch of Macromiidae does not cluster with other Anisoptera. S. fonscolombii is far decoupled from other Sympetrum spp. We may conclude that the ITS region is unable to adequately resolve the phylogeny at the level of Odonata families

**Fig. 6.**
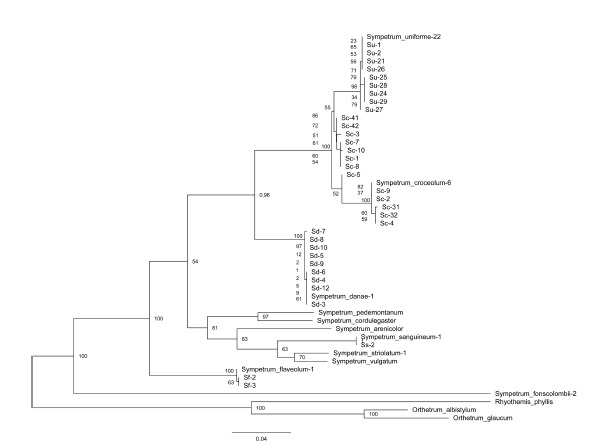
Phylogenetic tree of the studied Sympetrum species reconstructed with the Maximum Likelihood method from the histone H3–H4 region sequences. Bootstrap values are shown at the respective nodes. Orthetrum albistylum, O. glaucum and Rhyothemis phyllis serve as outgroup.

To test the applicability of the proposed marker, the histone H3–H4 region, to evaluating intra-generic and intra-species variation, we estimated its variation and reconstructed a phylogenetic tree for 45 specimens belonging to 11 species of the genus Sympetrum involved, using the sequences of Rhyothemis phyllis (Sulzer, 1776), Orthetrum albistylum (Selys, 1848) and O. glaucum (Brauer, 1865) as the outgroup (Fig. 6). The magnitude of intra-species variation of the histone H3–H4 region sequence appeared quite substantial. For the three species represented by 10 to 14 specimens, S. croceolum, S. uniforme and S. danae, the maximum uncorrected p-distances (that is, the share of variable positions among all positions) between different alleles within a species appeared to be respectively 0.0216, 0.0037 and 0.0011, that is ca 2.1, 0.4 and 0 %. The averaged differences between any two sequences within S. croceolum, S. uniforme and S. danae were 0.0165, 0.0009 and 0.0003, respectively

In the reconstructed phylogenetic tree (Fig. 6), ten sequences of S. uniforme, ten sequences of S. danae and three sequences of S. flaveolum expectedly clustered with the maximum bootstrap support of 100. Strikingly, the cluster of S. uniforme appeared to be nested inside that of S. croceolum, with the united cluster of these two species also having the support of 100. S. pedemontanum (Müller in Allioni, 1766) and S. cordulegaster clustered with a support of 76. At the same time, S. fonscolombii showed a very deep divergence from the rest of Sympetrum. We may conclude that sequences of different specimens of a species clustered together with the maximum support or nearly so, and cases of tight clustering of different species corresponded to the notion of their relatedness based on morphology

It is noteworthy that the sequences of two specimens of S. croceolum from its West Siberian isolate (specimens Sc-41 and Sc-42) (Kosterin, 2002) did not show divergence from those from the main Far Eastern range of the species (the rest of the specimens) but were nested among them

## Discussion

**The marker proposed**

We may conclude that the phylogenetic information provided by the proposed marker well resolved the overall phylogenetic relationships of Odonata at taxonomic levels of families, genera and species (Fig. 2), with few notable exceptions, which could actually reflect weak points of the currently accepted taxonomic system (Dijkstra et al., 2013).

The high conservation of the histone H3 and H4 proteins is paralleled by the high conservation of their coding sequences, the variation of which is nearly confined to synonymous substitutions (Stein et al., 1984; Doenecke et al., 1997; Eirín- López et al., 2009). This allowed us to design primers highly specific to these particular genes but of a very broad applicability to biological objects. It is noteworthy that the substitutions in the histone coding sequence, the overwhelming majority of which are synonymous, provide enough variation for satisfactory resolution of phylogenetic relations between the studied species (Fig. 3). Because of this, the histone H3 gene coding sequence has been broadly used as a phylogenetic marker for short evolutionary distances, e. g. in Odonata by Carle et al. (2015), with conserved positions permitting universal primers whereas the phylogenetic signal being mostly provided by synonymous substitutions.

The marker proposed here, the histone H3–H4 region, has an advantage of possibility to design highly universal primers matching the most conserved eukaryotic coding regions, those of histones H3 and H4. Note that we used the LH4_2R primer designed to match the sequence of a lepidopteran, B. mori. The amplified fragment contains most of the coding region of histone H4 and about a half of that of histone H3, and ca 250 b.p. long spacer between them. No significant adaptive constraint is expected for variation of the spacer, except for the origins of transcription of both genes (in opposite directions, with the transcribed sequences not overlapping), which the spacer contains judging from transcriptome data in public databases (not shown). Hence, the spacer enjoys mostly a neutral regime of evolution and may serve as a molecular clock

The histone H3–H4 region includes both highly conserved coding sequences and a neutrally variable non-coding spacer, and is tandemly repeated; this makes the proposed phylogenetic marker similar in biological and technical respects to such a popular nuclear marker as the ITS region of the nucleolus organiser (for its use in Odonata, see Hovmöller, Johansson, 2004; Dumont et al., 2010; Schneider et al., 2023) including the internal spacers ITS1 and ITS2 and the functional 5.8 S rRNA between them. The length of ca 250 b.p. of the spacer in the marker proposed here is comparable to ca 200 b.p. of ITS1 and ca 160 b.p. of ITS2 (these figures are for Odonata). Both markers, the ITS and the histone H3–H4 region, have comparable lengths (ca 900 b.p.) and suffer from the same drawback of certain ambiguity of alignment because of frequent indels. Both are encoded by the nuclear genome but functionally unrelated. Hence the histone H3–H4 region can be used for the same purposes as the ITS region. Moreover, comparison of phylogenetic resolution of Odonata at the family level (Figs 2, 5) showed that the H3–H4 region adequately resolved the phylogeny of the Odonata families (Fig. 2) while the ITS region rather failed to do this (Fig. 5), so the use of the former is preferable at this level.

Therefore, the use of the histone H3–H4 region can update the traditional analysis of the ITS region with about the same amount of independent phylogenetic information of the same nature. A joint analysis of both similar but unrelated nuclear markers, the ITS and H3–H4 regions (by their concatenation or, better, involving software specially designed for simultaneous analysis of different markers), is expected to provide a more robust phylogenetic inference than the analysis of ITS alone. Judging by the phylogenetic trees obtained (Figs 2, 6), the use of the histone H3–H4 region as a phylogenetic marker is highly recommendable at the levels of species and genera. Since it correctly resolves the family structure of the order, with few exceptions, it could also be useful at the level of families as well, but better as an additional marker analysed together with other phylogenetic markers

**Applicability of the new marker beyond Odonata**

Because of conservativeness of the histone H3 and H4 genes, the new marker can be used with the primers provided herein for any Odonata and other insects with the same order and orientation of the histone H3 and H4 genes in the histone repeat. Our investigation of public databases revealed the same order and orientation of these two genes in the histone repeat in a number of species of Diptera. In the present study, they are exemplified by such genera as Aedes Meigen, 1818 and Anopheles Meigen, 1818, used as the outgroup in the phylogenetic trees of Figures 2–4. The same order was found in D. melanogaster (Goodenough, 1984), which represents another suborder of Diptera. Besides, the same was found in the Formica Linnaeus, 1758 ants representing Hymenoptera.

For the use of the marker proposed here in insects with other order or orientations of these genes, other relevant primers have to be worked out. For example, in Lepidoptera, where the genes of histones H3 and H4 have parallel orientation, the same LH4-2R primer can be used in combination with a primer of the sequence which is a reverse complement of that of the Hex-AR primer. In this case, the portion of the coding sequence of the H3 gene will be smaller, 37 b.p., and the spacer will be somewhat longer – about 900–1400 b.p.

Histone genes are organised in tandem repeats in a broad range of large groups of organisms such as amphibians, fish, echinoderms, arthropods and annelids (Eirín-López et al., 2009). Among the examples given by Eirín-López et al. (2009), beyond insects, the genes of histones H3 and H4 are adjacent in the histone repeat in the rainbow trout (fish), Xenopus spp. (amphibians), starfish (species not indicated, echinoderms), Asellus aquaticus (Linnaeus, 1758) (crustacean) and three species from different genera of annelids (Eirín-López et al., 2009: Fig. 8.2). All these groups are potential targets for the use of versions of the marker proposed here based on the coding sequences of the histone H3 and H4 genes and the spacer between them, taking into account orientation of the two genes. Moreover, among the organisms mentioned, the crustacean A. aquaticus and the annelids Platynereis dumerilii (Audouin, Milne-Edwards, 1834) and Chaetopterus variopedatus (Reiner, 1804) have the same orientation of the two genes as Odonata (Eirín-López et al., 2009: Fig. 8.2). Taking into account the high conservativeness of the histone H3 and H4 genes, it is not excluded that the marker proposed here is applicable to these objects with the same primers.

**Phylogenetic relationships among Sympetrum spp.**

**Fig. 7.**
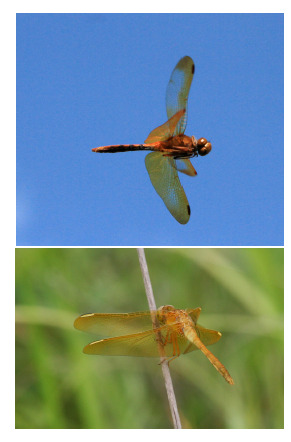
Males of Sympetrum croceolum (top – Russia, Novosibirsk Academy Town env., 24.08.2023) and S. uniforme (bottom – Russia, Primorye, Gornye Klyuchi env., Draguchina Arm of the Ussuri River, 30.07.2020) in nature. Photos by O. Kosterin

The analysis of the new marker yielded two rather unexpected results at once, both concerning the species S. croceolum and S. uniforme (Fig. 6). First, S. uniforme appeared to be an inner branch nested inside S. croceolum. This result appeared robust regardless of the methods and models of phylogenetic reconstructions (not shown). These species are obviously related but well distinguishable by the morphology of the male genitalia and female vulvar scale, wing coloration (dull, complete but gradually changing in intensity in S. uniforme versus bright but with gaps in S. croceolum) (Fig. 7) and size (in the former it is somewhat larger). In East Asia, they usually co-exist in a wide range of lentic habitats (Onishko, Kosterin, 2021), while S. croceolum also has an isolated range fragment in the southern West Siberia (Kosterin, 1987; 2002; Popova, Haritonov, 2020). It should be stressed that specimens of S. uniforme, identified by external characters, formed a highly supported cluster (Fig. 6). There is no doubt that S. croceolum and S. uniforme are bona species. The phylogenetic pattern obtained, where the sequences of S. uniforme are nested inside those of S. croceolum, suggests S. uniforme being a phyletic descendant of S. croceolum, which hence appeared paraphyletic. This contradicts the cladistic approach in systematics and the phylogenetic concept of species. At the same time, this pattern fits well the so-called punctuated equilibria mode of speciation (Eldredge, Gould, 1972), suggesting that speciation takes place for short time periods in evolutionary scale (tens to hundreds of thousand years) in small, isolated populations in the periphery of parental species’ ranges, while species exist almost unchanged for millions of years (evolutionary stasis). This concept better fits the basics of evolutionary genetics (Mayr, 1963; Berdnikov, 1999) than the earlier prevailing model of gradual divergent evolution. In the punctuated evoltion point of view, species ‘propagate’ as if being individuals, with younger species often co-existing with their parental species.

In the phylogenetic tree based on the histone H3–H4 region (Fig. 6), two analyzed specimens of S. croceolum from its West Siberian isolate (specimens Sc-41 and Sc-42 from Lake Manzherok) lacked supported divergence from specimens from the main Far Eastern range of the species (the rest of the specimens), which is quite remarkable. Specimens from the West Siberian isolate (Kosterin, 2002) differ from the Far Eastern specimens by a much more developed wing amber colour and an appearance of a brown infumation in the wing apical parts and so were for a long time supposed to represent a separate subspecies (Kosterin, 1987; Popova, Haritonov, 2020), which, however, has not been named yet. This lack of divergence at the molecular level suggests the West Siberian isolate to be very young in evolutionary time scale and well fits its hypothetic Holocene age, implying the range split after Atlantic time (Kosterin, 2002), as well as in some nemoral species of Lepidoptera (Dubatolov, Kosterin, 2000; Solovyev et al., 2015, 2022). This, however, does not exclude a subspecies rank of the West Siberian population(s), since subspecies are entities of well-defined geographical variation for some phenotype characters, which implies specific divergence of the genes determining these characters rather than those of the entire genome

In all phylogenetic reconstructions from sequences of the histone H3–H4 region (Figs 2, 6), S. fonscolombii is strongly diverged from the rest of the genus Sympetrum. The same result was earlier obtained by Pilgrim and von Dohlen (2012) who undertook a molecular phylogenetic study of Sympetrum and related genera based on the joint analysis of the nuclear marker EF-1α and ITS2, and the mitochondrial genes 16S, tRNA valine, 12S, and COI. This divergent position of S. fonscolombii has long ago been recognised at the level of phenotype, resulting in a suggestion to move S. fonscolombii to the genus Tarnetrum Needham and Fischer, 1936 (Schmidt, 1987). This genus was erected for two Nearctic species, Mesothemis corrupta Hagen 1861, and M. illota Hagen, 1861 (mentioned in (Schmidt, 1987) as well as presently considered in combinations S. illotum and S. corruptum), with M. illota (sub. S. illotum) indicated as the type species (Needham, Fischer, 1936). However, according to Pilgrim and von Dohlen (2012), S. corruptum is quite closely related to S. fonscolombii (together with S. villosum Ris, 1911 and Nesogonia blackburni (McLachlan, 1883)) while S. illotum is not. Since the type species of the genus Tarnetrum is not closely related to S. fonscolombii, this genus is not suitable for the latter species (Dijkstra, Kalkman, 2015). Therefore, the genus Sympetrum in the current sense deserves further reconsideration, maybe with erection of a new genus at least for the fonscolombii-group sensu Pilgrim et al. (2012).

## Conflict of interest

The authors declare no conflict of interest.
